# Crystal structure of aqua[*N*-(2-oxidobenzyl-κ*O*)-l-leucinato-κ^2^ *N*,*O*](1,10-phenanthroline-κ^2^ *N*,*N*′)­nickel(II) penta­hydrate

**DOI:** 10.1107/S2056989015001085

**Published:** 2015-01-21

**Authors:** Md. Serajul Haque Faizi, Natalia O. Sharkina

**Affiliations:** a Department of Chemistry, Indian Institute of Technology Kanpur, Kanpur, UP 208 016, India; b National Taras Shevchenko University, Department of Chemistry, Volodymyrska str. 64, 01601 Kyiv, Ukraine

**Keywords:** crystal structure, nickel(II) complex, hydrogen bonding

## Abstract

In the title compound, [Ni(C_13_H_17_NO_3_)(C_12_H_8_N_2_)(H_2_O)]·5H_2_O, the Ni^II^ atom is in a distorted octa­hedral coordination environment provided by the two N atoms of one bidentate phenanthroline ligand and two O atoms and one N atom from a tridentate 2-[(2-hy­droxy­benz­yl)amino]-4-methyl­penta­noic acid (HAMA) ligand and one water mol­ecule. The complex was prepared by the reaction of nickel(II) nitrate with HAMA in the presence of 1,10-phenanthroline in a 1:1:1 ratio. In the crystal, the complex mol­ecules and solvate water mol­ecules are associated *via* O—H⋯O hydrogen bonds into a three-dimensional network.

## Abstract 2

The Ni^II^ atom in the title compound is in a distorted octa­hedral coordination geometry with two N atoms of the phenanthroline ligand, two O and one N atom of the 2-[(2-hy­droxy­benz­yl)amino]-4-methyl­penta­noic acid ligand and one water mol­ecule. In the crystal, the complex mol­ecules and solvate water mol­ecules are associated *via* O—H⋯O hydrogen bonds into a three-dimensional network.

## Chemical context   

Metal complexes of 1,10-phenanthroline (phen) and its deriv­atives are of increasing inter­est because of their versatile roles in many fields such as analytical chemistry (Chalk & Tyson, 1994 ▸), catalysis (Samnani *et al.*, 1996 ▸), electrochemical polymerization (Bachas *et al.*, 1997 ▸), and biochemistry (Sammes & Yahioglu, 1994 ▸). 1,10-Phenanthroline is a chelating bidentate ligand with notable coordination ability for transition metal cations. It is widely used in coordination chemistry, in particular, for the preparation of mixed-ligand complexes (Fritsky *et al.*, 2004 ▸; Kanderal *et al.*, 2005 ▸), and in the synthesis of polynuclear complexes and coordination polymers in order to control nuclearity and dimensionality by blocking a certain number of vacant sites in the coordination sphere of a metal ion (Fritsky *et al.*, 2006 ▸; Penkova *et al.*, 2010 ▸). Over the last few decades, the complex formation of transition metal ions with amino acids has also been studied extensively (Auclair *et al.*, 1984 ▸). Amino acid–metallic ion inter­actions are found to be responsible for enzymatic activity and the stability of protein structures (Dinelli *et al.*, 2010 ▸). Nickel is also essential for the healthy life of animals. It is associated with several enzymes (Poellot *et al.*, 1990 ▸) and plays a role in physiological processes as a co-factor in the absorption of iron from the intestine (Nielsen *et al.*, 1980 ▸). Any change in its concentration leads to metabolic disorder (Kolodziej, 1994 ▸). With the discovery of the biological importance of nickel, it is essential to study its complex formation with amino acids in order to understand more about the functions of their complexes.

## Structural commentary   

**Figure 1:**
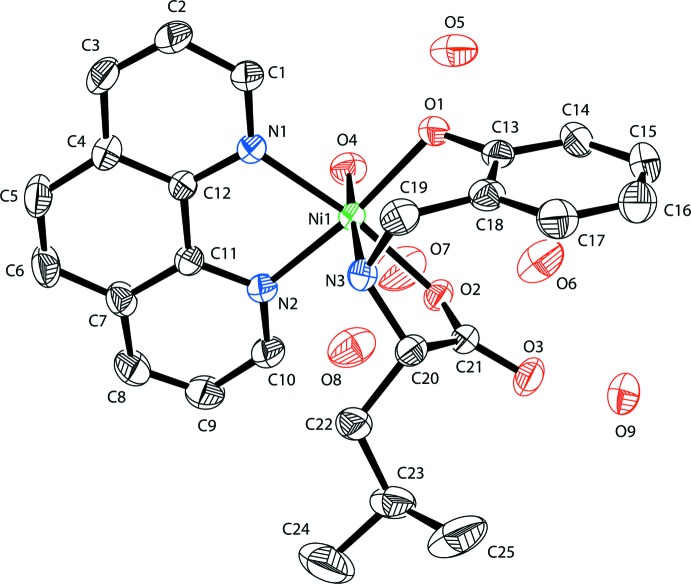
The mol­ecular structure and atom-numbering scheme for the title compound, with displacement ellipsoids drawn at the 40% probability level.

The Ni^II^ ion in the title compound is in a distorted octa­hedral coordination environment provided by the two N atoms of one bidentate phen ligand and two O atoms and one N atom from a tridentate anion of HAMA and one water mol­ecule (Fig. 1 ▸).
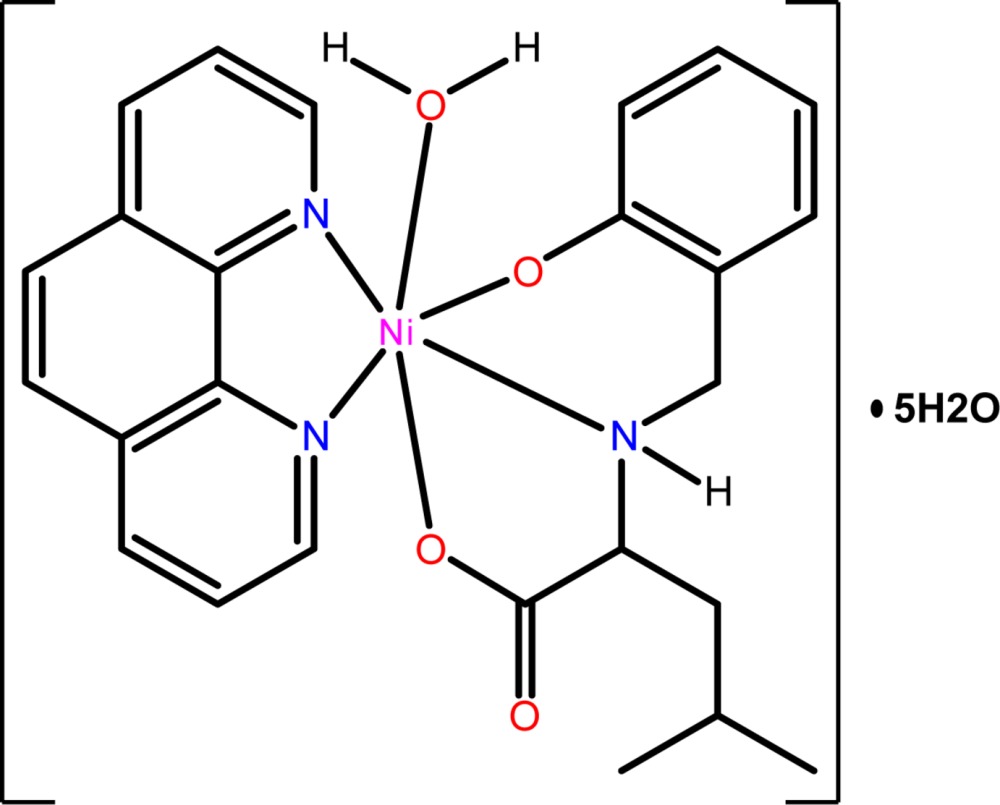


 The equatorial plane consists of two nitro­gen atoms of 1,10-phenanthroline and two oxygen atoms of the HAMA ligand. The axial positions are occupied by the nitro­gen atom from the HAMA ligand and a water O atom. The equatorial Ni—N and Ni—O bond lengths are in the range 2.0383 (11)– 2.1058 (13) Å, the axial Ni—N and Ni—O bond lengths are 2.1429 (14) and 2.1110 (12) Å. The coordination Ni—N and Ni—O bond lengths are typical for distorted octa­hedral Ni^II^ complexes with nitro­gen and oxygen donors (Fritsky *et al.*, 1998 ▸; Moroz *et al.*, 2012 ▸). The N1—Ni1—N2 and O2—Ni1—N3 bite angles are decreased to 79.43 (5) and 80.50 (5)° as a consequence of the formation of the five-membered chelate rings. The C—C and C—N bond lengths in the organic ligands are well within the limits expected for those in aromatic rings (Petrusenko *et al.*, 1997 ▸; Strotmeyer *et al.*, 2003 ▸; Penkova *et al.*, 2009 ▸).

## Supra­molecular features   

**Figure 2:**
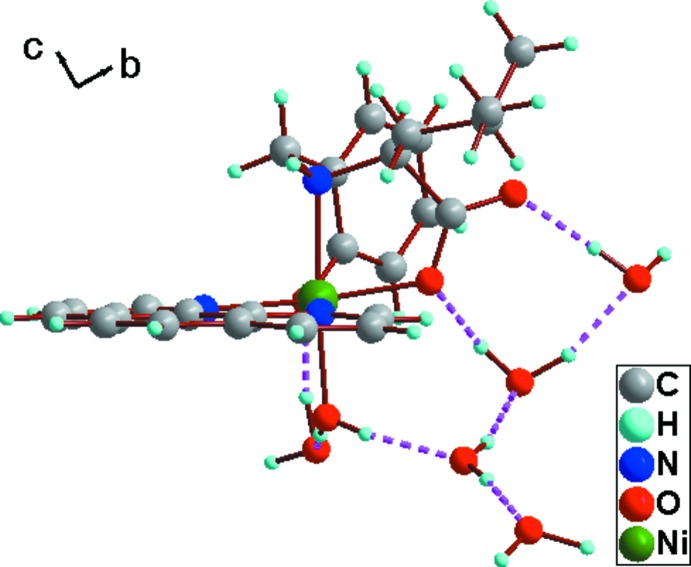
A view of the O—H⋯O hydrogen-bond inter­actions between the donor atoms of the water mol­ecules and acceptor oxygen atoms of the phenolic and carb­oxy­lic groups and solvate water mol­ecules in the crystal of the title compound (hydrogen bonds are shown as dashed lines; see Table 1 ▸ for details).

**Figure 3:**
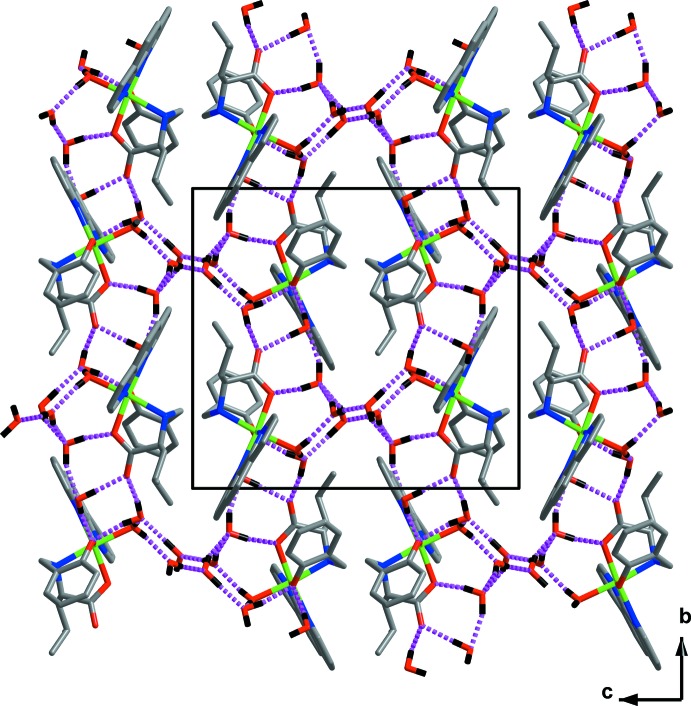
A view along the *a* axis of the crystal packing of the title compound. The O—H⋯O hydrogen-bonding inter­actions between the donor atoms of the water mol­ecules and acceptor oxygen atoms of the phenolic and carb­oxy­lic groups and solvate water mol­ecules are shown as magenta dashed lines (see Table 1 ▸ for details).

In the crystal packing, the complex mol­ecules and solvate water mol­ecules are associated *via* inter­molecular hydrogen bonds (Table 1 ▸ and Fig. 2 ▸) that involve O—H inter­actions of medium strength between the donor atoms of the water mol­ecules and acceptor oxygen atoms of the phenolic and the carb­oxy­lic groups and solvate water mol­ecules, forming a three-dimensional network (Fig. 3 ▸).

## Synthesis and crystallization   

The ligand 2-[(2-hy­droxy­benz­yl)amino]-4-methyl­penta­noic acid (HAMA) was prepared by following procedure: l-Leucine (1.00 g, 6.71 mmol) and LiOH·H_2_O (0.284 g, 6.77 mmol) in dry methanol (30 ml) were stirred for 30 min to dissolve. A methano­lic solution of salicyl­aldehyde ­(1.44 g, 6.72 mmol) was added dropwise to the above solution. The solution was stirred for 1 h and then treated with sodium borohydride (0.248 g, 6.71 mmol) with constant stirring. The solvent was evaporated and the resulting sticky mass was dissolved in water. A cloudy solution was obtained, which was then acidified with dilute HCl and the solution pH was maintained between 5–7. The ligand precipitated as a colourless solid. The solid was filtered off, thoroughly washed with water and finally dried inside a vacuum desiccator. Yield 2.08 g (85%).

The title compound was prepared as follows: HAMA (0.500 g, 1.43 mmol) was deprotonated with LiOH·H_2_O (0.060 g, 1.44 mmol) in 25 ml MeOH, which resulted in a clear colourless solution after 30 min. A methano­lic solution of Ni(NO_3_)_2_·6H_2_O (0.17 g, 0.71 mmol) was added dropwise to the ligand with stirring. The colour of the solution changed to green immediately. The solution was stirred for 2 h and evaporated to dryness on a rotary evaporator. The blue solid obtained by adding aceto­nitrile was recrystallized as green plates by slow diffusion of diethyl ether into a methano­lic solution of the crude solid over 2–3 days. The crystals were filtered off and washed with diethyl ether. Yield 74%.

## Refinement   

Crystal data, data collection and structure refinement details are summarized in Table 2 ▸. The N—H hydrogen atoms were located in a difference Fourier map and freely refined. The O—H hydrogen atoms were also located in a difference Fourier map but constrained to ride on their parent atoms with *U*_iso_(H) = 1.5*U*_eq_(O). The C-bound H atoms were included in calculated positions and treated as riding atoms: with C—H = 0.95 Å and *U*_iso_(*H*) = 1.2–1.5*U*_eq_(C).

## Supplementary Material

Crystal structure: contains datablock(s) global, I. DOI: 10.1107/S2056989015001085/ff2134sup1.cif

Structure factors: contains datablock(s) I. DOI: 10.1107/S2056989015001085/ff2134Isup2.hkl

CCDC reference: 1044097

Additional supporting information:  crystallographic information; 3D view; checkCIF report

## Figures and Tables

**Table 1:** Hydrogen-bond geometry (, )

*D*H*A*	*D*H	H*A*	*D* *A*	*D*H*A*
O4H1*O*4O7	0.83	1.89	2.709(2)	169
O4H2*O*4O5	0.98	1.80	2.772(2)	169
O5H1*O*5O3^i^	1.00	1.82	2.8137(19)	171
O5H2*O*5O1	0.95	1.81	2.7393(19)	164
O6H1*O*6O2	0.96	1.83	2.7310(18)	156
O6H2*O*6O9	1.00	1.85	2.807(2)	160
O7H1*O*7O8	0.93	1.78	2.693(3)	171
O7H2*O*7O6	0.94	1.91	2.832(3)	165
O8H1*O*8O6^ii^	0.87	1.89	2.730(3)	162
O8H2*O*8O5^ii^	1.08	1.68	2.749(2)	169
O9H1*O*9O3	0.95	1.81	2.749(2)	171
O9H2*O*9O1^iii^	0.87	1.98	2.8459(18)	173

Symmetry codes: (i) 
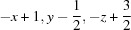
; (ii) 
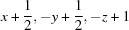
; (iii) 
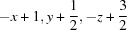
.

**Table 2:** Experimental details

Crystal data	
Chemical formula	[Ni(C_13_H_17_NO_3_)(C_12_H_8_N_2_)(H_2_O)]5H_2_O
*M*_r_	582.29
Crystal system, space group	Orthorhombic, *P*2_1_2_1_2_1_
Temperature (K)	100
*a*, *b*, *c* ()	11.7968(2), 14.8290(3), 16.1406(3)
*V* (^3^)	2823.55(9)
*Z*	4
Radiation type	Mo *K*
(mm^1^)	0.74
Crystal size (mm)	0.30 0.21 0.15

Data collection	
Diffractometer	Bruker SMART APEX CCD
Absorption correction	Multi-scan (*SADABS*; Bruker, 2001 ▸)
*T*_min_, *T*_max_	0.803, 0.865
No. of measured, independent and observed [*I* > 2(*I*)] reflections	29541, 5240, 5046
*R*_int_	0.024
(sin /)_max_ (^1^)	0.606

Refinement	
*R*[*F* ^2^ > 2(*F* ^2^)], *wR*(*F* ^2^), *S*	0.022, 0.057, 1.03
No. of reflections	5240
No. of parameters	347
H-atom treatment	H atoms treated by a mixture of independent and constrained refinement
_max_, _min_ (e ^3^)	0.22, 0.24
Absolute structure	(Flack, 1983 ▸), 2291 Friedel pairs
Absolute structure parameter	0.008(7)

Computer programs: *SMART* and *SAINT* (Bruker, 2003 ▸), *SIR97* (Altomare *et al.*, 1999 ▸), *SHELXL97* (Sheldrick, 2015 ▸) and *DIAMOND* (Brandenburg Putz, 2006 ▸).

## supplementary crystallographic information

### Crystal data

**Table:**

[Ni(C_13_H_17_NO_3_)(C_12_H_8_N_2_)(H_2_O)]·5H_2_O	*F*(000) = 1232
*M**_r_* = 582.29	*D*_x_ = 1.370 Mg m^−^^3^
Orthorhombic, *P*2_1_2_1_2_1_	Mo *K*α radiation, λ = 0.71073 Å
Hall symbol: P 2ac 2ab	Cell parameters from 1399 reflections
*a* = 11.7968 (2) Å	θ = 2.6–28.6°
*b* = 14.8290 (3) Å	µ = 0.74 mm^−^^1^
*c* = 16.1406 (3) Å	*T* = 100 K
*V* = 2823.55 (9) Å^3^	Block, green
*Z* = 4	0.30 × 0.21 × 0.15 mm

### Data collection

**Table:**

Bruker SMART APEX CCD diffractometer	5240 independent reflections
Radiation source: fine-focus sealed tube	5046 reflections with *I* > 2σ(*I*)
Graphite monochromator	*R*_int_ = 0.024
ω scans	θ_max_ = 25.5°, θ_min_ = 2.9°
Absorption correction: multi-scan (*SADABS*; Bruker, 2001)	*h* = −14→14
*T*_min_ = 0.803, *T*_max_ = 0.865	*k* = −17→17
29541 measured reflections	*l* = −19→19

### Refinement

**Table:**

Refinement on *F*^2^	Secondary atom site location: difference Fourier map
Least-squares matrix: full	Hydrogen site location: inferred from neighbouring sites
*R*[*F*^2^ > 2σ(*F*^2^)] = 0.022	H atoms treated by a mixture of independent and constrained refinement
*wR*(*F*^2^) = 0.057	*w* = 1/[σ^2^(*F*_o_^2^) + (0.0322*P*)^2^ + 0.3183*P*] where *P* = (*F*_o_^2^ + 2*F*_c_^2^)/3
*S* = 1.03	(Δ/σ)_max_ = 0.001
5240 reflections	Δρ_max_ = 0.22 e Å^−^^3^
347 parameters	Δρ_min_ = −0.24 e Å^−^^3^
0 restraints	Absolute structure: (Flack, 1983), 2291 Friedel pairs
Primary atom site location: structure-invariant direct methods	Absolute structure parameter: 0.008 (7)

### Special details

**Table:**

Geometry. All esds (except the esd in the dihedral angle between two l.s. planes) are estimated using the full covariance matrix. The cell esds are taken into account individually in the estimation of esds in distances, angles and torsion angles; correlations between esds in cell parameters are only used when they are defined by crystal symmetry. An approximate (isotropic) treatment of cell esds is used for estimating esds involving l.s. planes.
Refinement. Refinement of F^2^ against ALL reflections. The weighted R-factor wR and goodness of fit S are based on F^2^, conventional R-factors R are based on F, with F set to zero for negative F^2^. The threshold expression of F^2^ > 2sigma(F^2^) is used only for calculating R-factors(gt) etc. and is not relevant to the choice of reflections for refinement. R-factors based on F^2^ are statistically about twice as large as those based on F, and R- factors based on ALL data will be even larger.

### Fractional atomic coordinates and isotropic or equivalent isotropic displacement parameters (Å^2^)

**Table:**

	*x*	*y*	*z*	*U*_iso_*/*U*_eq_	
C1	0.67912 (16)	0.00202 (12)	0.88437 (13)	0.0487 (5)	
H1	0.6018	0.0137	0.8809	0.058*	
C2	0.71452 (18)	−0.07817 (13)	0.92143 (13)	0.0558 (5)	
H2	0.6614	−0.1186	0.9422	0.067*	
C3	0.82686 (17)	−0.09691 (13)	0.92708 (13)	0.0522 (5)	
H3	0.8514	−0.1496	0.9526	0.063*	
C4	0.90610 (15)	−0.03549 (12)	0.89372 (11)	0.0422 (4)	
C5	1.02594 (17)	−0.04941 (14)	0.89504 (13)	0.0553 (5)	
H5	1.0550	−0.1010	0.9200	0.066*	
C6	1.09760 (17)	0.01069 (15)	0.86086 (14)	0.0573 (5)	
H6	1.1752	−0.0002	0.8625	0.069*	
C7	1.05636 (15)	0.09111 (13)	0.82204 (11)	0.0442 (4)	
C8	1.12618 (17)	0.15465 (15)	0.78282 (14)	0.0576 (5)	
H8	1.2044	0.1468	0.7819	0.069*	
C9	1.07804 (18)	0.22823 (16)	0.74592 (15)	0.0589 (5)	
H9	1.1231	0.2701	0.7184	0.071*	
C10	0.96122 (16)	0.24002 (13)	0.74975 (13)	0.0481 (4)	
H10	0.9299	0.2907	0.7246	0.058*	
C11	0.93931 (14)	0.10790 (11)	0.82141 (9)	0.0346 (3)	
C12	0.86278 (14)	0.04341 (10)	0.85744 (10)	0.0339 (3)	
C13	0.48304 (14)	0.24458 (11)	0.83796 (11)	0.0386 (4)	
C14	0.39435 (15)	0.28423 (13)	0.79308 (14)	0.0514 (4)	
H14	0.3766	0.2628	0.7405	0.062*	
C15	0.33276 (18)	0.35528 (15)	0.82648 (16)	0.0643 (6)	
H15	0.2743	0.3811	0.7959	0.077*	
C16	0.35715 (19)	0.38806 (15)	0.90432 (16)	0.0649 (6)	
H16	0.3146	0.4348	0.9269	0.078*	
C17	0.44555 (17)	0.35059 (14)	0.94819 (13)	0.0539 (5)	
H17	0.4632	0.3735	1.0002	0.065*	
C18	0.50909 (15)	0.27931 (12)	0.91669 (11)	0.0415 (4)	
C19	0.60869 (16)	0.24162 (13)	0.96300 (11)	0.0462 (4)	
H19A	0.6129	0.2693	1.0174	0.055*	
H19B	0.5987	0.1772	0.9705	0.055*	
C20	0.73468 (15)	0.35627 (11)	0.90083 (11)	0.0388 (4)	
H20	0.6897	0.3914	0.9404	0.047*	
C21	0.69476 (13)	0.37869 (10)	0.81280 (11)	0.0362 (3)	
C22	0.86072 (17)	0.37934 (13)	0.91303 (12)	0.0471 (4)	
H22A	0.8783	0.3737	0.9715	0.056*	
H22B	0.9055	0.3346	0.8838	0.056*	
C23	0.89868 (19)	0.47278 (15)	0.88411 (13)	0.0598 (6)	
H23	0.8787	0.4785	0.8254	0.072*	
C24	1.0279 (3)	0.4786 (2)	0.8908 (2)	0.1145 (13)	
H24A	1.0527	0.5372	0.8730	0.172*	
H24B	1.0617	0.4333	0.8563	0.172*	
H24C	1.0504	0.4691	0.9473	0.172*	
C25	0.8371 (3)	0.54780 (16)	0.93128 (17)	0.0957 (10)	
H25A	0.8623	0.6054	0.9113	0.144*	
H25B	0.8536	0.5428	0.9893	0.144*	
H25C	0.7569	0.5422	0.9227	0.144*	
N1	0.75051 (11)	0.06215 (9)	0.85396 (9)	0.0368 (3)	
N2	0.89272 (11)	0.18292 (10)	0.78726 (8)	0.0370 (3)	
N3	0.71624 (14)	0.25874 (9)	0.91724 (9)	0.0373 (3)	
H1N3	0.7731 (17)	0.2423 (12)	0.9458 (11)	0.035 (5)*	
O1	0.54218 (9)	0.17497 (7)	0.80691 (8)	0.0404 (2)	
O2	0.70273 (9)	0.31677 (7)	0.75881 (7)	0.0378 (2)	
O3	0.66098 (12)	0.45636 (8)	0.79805 (9)	0.0528 (3)	
O4	0.71500 (12)	0.12784 (8)	0.68295 (7)	0.0482 (3)	
H1O4	0.7442	0.1593	0.6462	0.072*	
H2O4	0.6364	0.1109	0.6697	0.072*	
O5	0.48550 (14)	0.09831 (10)	0.65816 (9)	0.0655 (4)	
H1O5	0.4369	0.0440	0.6693	0.098*	
H2O5	0.4945	0.1184	0.7136	0.098*	
O6	0.59572 (17)	0.35179 (11)	0.61226 (9)	0.0762 (5)	
H1O6	0.6134	0.3321	0.6675	0.114*	
H2O6	0.5949	0.4193	0.6121	0.114*	
O7	0.78670 (19)	0.24699 (12)	0.56707 (11)	0.0893 (6)	
H1O7	0.8508	0.2601	0.5362	0.134*	
H2O7	0.7207	0.2824	0.5720	0.134*	
O8	0.96331 (17)	0.27411 (12)	0.46430 (12)	0.0867 (6)	
H1O8	1.0055	0.2280	0.4505	0.130*	
H2O8	0.9617	0.3251	0.4163	0.130*	
O9	0.61321 (12)	0.53645 (10)	0.64845 (9)	0.0572 (4)	
H1O9	0.6214	0.5095	0.7015	0.086*	
H2O9	0.5648	0.5795	0.6578	0.086*	
Ni1	0.715392 (16)	0.187826 (13)	0.801560 (13)	0.03288 (6)	

### Atomic displacement parameters (Å^2^)

**Table:**

	*U*^11^	*U*^22^	*U*^33^	*U*^12^	*U*^13^	*U*^23^
C1	0.0358 (9)	0.0380 (10)	0.0724 (13)	−0.0048 (7)	0.0006 (8)	0.0138 (9)
C2	0.0498 (11)	0.0403 (10)	0.0772 (13)	−0.0072 (9)	0.0056 (11)	0.0187 (9)
C3	0.0570 (12)	0.0343 (9)	0.0651 (12)	0.0052 (8)	0.0014 (9)	0.0155 (9)
C4	0.0454 (10)	0.0341 (9)	0.0471 (9)	0.0074 (7)	−0.0011 (8)	0.0031 (7)
C5	0.0485 (11)	0.0520 (12)	0.0654 (12)	0.0192 (9)	0.0002 (9)	0.0140 (9)
C6	0.0355 (10)	0.0655 (13)	0.0708 (13)	0.0179 (9)	0.0033 (9)	0.0117 (11)
C7	0.0328 (9)	0.0484 (10)	0.0514 (10)	0.0053 (7)	0.0029 (7)	0.0009 (8)
C8	0.0319 (9)	0.0648 (13)	0.0759 (14)	−0.0012 (9)	0.0116 (9)	0.0071 (10)
C9	0.0448 (11)	0.0580 (12)	0.0738 (14)	−0.0115 (10)	0.0132 (10)	0.0113 (11)
C10	0.0455 (10)	0.0387 (10)	0.0603 (11)	−0.0033 (8)	0.0030 (9)	0.0095 (8)
C11	0.0348 (8)	0.0328 (8)	0.0361 (8)	0.0013 (6)	0.0010 (6)	−0.0019 (6)
C12	0.0335 (8)	0.0292 (8)	0.0390 (8)	0.0017 (7)	−0.0006 (7)	−0.0018 (6)
C13	0.0296 (8)	0.0320 (8)	0.0543 (10)	−0.0035 (7)	0.0063 (7)	−0.0025 (7)
C14	0.0353 (9)	0.0514 (10)	0.0675 (12)	0.0012 (8)	−0.0067 (9)	−0.0073 (9)
C15	0.0373 (10)	0.0563 (12)	0.0992 (19)	0.0117 (9)	−0.0065 (10)	−0.0017 (12)
C16	0.0484 (12)	0.0530 (12)	0.0934 (17)	0.0088 (10)	0.0168 (12)	−0.0158 (12)
C17	0.0493 (11)	0.0539 (11)	0.0586 (12)	−0.0010 (9)	0.0143 (9)	−0.0095 (9)
C18	0.0381 (9)	0.0401 (9)	0.0463 (9)	−0.0054 (7)	0.0103 (7)	0.0008 (8)
C19	0.0523 (11)	0.0459 (10)	0.0403 (9)	−0.0040 (8)	0.0057 (8)	0.0054 (8)
C20	0.0435 (10)	0.0311 (8)	0.0418 (9)	−0.0003 (7)	−0.0042 (7)	−0.0003 (7)
C21	0.0347 (8)	0.0277 (8)	0.0462 (9)	0.0002 (6)	−0.0044 (7)	0.0027 (7)
C22	0.0501 (11)	0.0431 (10)	0.0481 (10)	−0.0064 (8)	−0.0141 (8)	0.0010 (8)
C23	0.0708 (14)	0.0579 (13)	0.0508 (11)	−0.0251 (11)	−0.0197 (10)	0.0101 (9)
C24	0.0807 (19)	0.118 (3)	0.145 (3)	−0.0561 (19)	−0.045 (2)	0.053 (2)
C25	0.161 (3)	0.0464 (13)	0.0799 (17)	−0.0191 (16)	−0.0274 (18)	−0.0082 (12)
N1	0.0331 (7)	0.0304 (7)	0.0469 (8)	0.0005 (5)	−0.0002 (5)	0.0050 (6)
N2	0.0345 (7)	0.0322 (7)	0.0443 (7)	−0.0020 (6)	0.0005 (5)	0.0034 (6)
N3	0.0368 (7)	0.0345 (7)	0.0407 (7)	0.0008 (7)	−0.0061 (7)	0.0070 (6)
O1	0.0325 (5)	0.0341 (6)	0.0545 (6)	−0.0012 (4)	−0.0014 (5)	−0.0060 (6)
O2	0.0436 (6)	0.0292 (5)	0.0405 (5)	0.0021 (6)	−0.0054 (5)	0.0037 (5)
O3	0.0646 (8)	0.0328 (6)	0.0611 (8)	0.0125 (6)	−0.0169 (7)	0.0003 (6)
O4	0.0507 (7)	0.0445 (7)	0.0496 (7)	−0.0037 (6)	0.0031 (6)	−0.0051 (5)
O5	0.0754 (10)	0.0614 (9)	0.0598 (8)	−0.0264 (8)	−0.0071 (7)	0.0030 (7)
O6	0.1227 (15)	0.0539 (9)	0.0519 (8)	−0.0023 (10)	−0.0210 (9)	0.0008 (7)
O7	0.1090 (14)	0.0725 (11)	0.0864 (12)	−0.0087 (11)	0.0442 (12)	0.0061 (9)
O8	0.1077 (15)	0.0609 (10)	0.0915 (12)	−0.0078 (10)	0.0424 (11)	−0.0001 (9)
O9	0.0575 (9)	0.0537 (8)	0.0603 (8)	0.0147 (7)	0.0051 (7)	0.0009 (7)
Ni1	0.02919 (10)	0.02618 (10)	0.04328 (10)	0.00102 (8)	−0.00074 (9)	0.00352 (9)

### Geometric parameters (Å, º)

**Table:**

C1—N1	1.321 (2)	C19—H19B	0.9700
C1—C2	1.395 (3)	C20—N3	1.486 (2)
C1—H1	0.9300	C20—C21	1.533 (2)
C2—C3	1.357 (3)	C20—C22	1.538 (3)
C2—H2	0.9300	C20—H20	0.9800
C3—C4	1.412 (3)	C21—O3	1.2417 (19)
C3—H3	0.9300	C21—O2	1.269 (2)
C4—C12	1.405 (2)	C22—C23	1.529 (3)
C4—C5	1.429 (3)	C22—H22A	0.9700
C5—C6	1.347 (3)	C22—H22B	0.9700
C5—H5	0.9300	C23—C24	1.531 (4)
C6—C7	1.432 (3)	C23—C25	1.531 (4)
C6—H6	0.9300	C23—H23	0.9800
C7—C8	1.402 (3)	C24—H24A	0.9600
C7—C11	1.403 (2)	C24—H24B	0.9600
C8—C9	1.367 (3)	C24—H24C	0.9600
C8—H8	0.9300	C25—H25A	0.9600
C9—C10	1.390 (3)	C25—H25B	0.9600
C9—H9	0.9300	C25—H25C	0.9600
C10—N2	1.318 (2)	N1—Ni1	2.0881 (14)
C10—H10	0.9300	N2—Ni1	2.1058 (13)
C11—N2	1.358 (2)	N3—Ni1	2.1429 (14)
C11—C12	1.438 (2)	N3—H1N3	0.849 (19)
C12—N1	1.354 (2)	O1—Ni1	2.0540 (11)
C13—O1	1.343 (2)	O2—Ni1	2.0383 (11)
C13—C14	1.402 (3)	O4—Ni1	2.1110 (12)
C13—C18	1.405 (3)	O4—H1O4	0.8299
C14—C15	1.389 (3)	O4—H2O4	0.9845
C14—H14	0.9300	O5—H1O5	1.0049
C15—C16	1.378 (3)	O5—H2O5	0.9485
C15—H15	0.9300	O6—H1O6	0.9604
C16—C17	1.378 (3)	O6—H2O6	1.0011
C16—H16	0.9300	O7—H1O7	0.9260
C17—C18	1.392 (3)	O7—H2O7	0.9419
C17—H17	0.9300	O8—H1O8	0.8742
C18—C19	1.501 (3)	O8—H2O8	1.0820
C19—N3	1.490 (2)	O9—H1O9	0.9494
C19—H19A	0.9700	O9—H2O9	0.8705
			
N1—C1—C2	122.94 (17)	C22—C20—H20	108.8
N1—C1—H1	118.5	O3—C21—O2	124.27 (16)
C2—C1—H1	118.5	O3—C21—C20	118.51 (15)
C3—C2—C1	119.71 (18)	O2—C21—C20	117.17 (13)
C3—C2—H2	120.1	C23—C22—C20	116.45 (17)
C1—C2—H2	120.1	C23—C22—H22A	108.2
C2—C3—C4	119.27 (17)	C20—C22—H22A	108.2
C2—C3—H3	120.4	C23—C22—H22B	108.2
C4—C3—H3	120.4	C20—C22—H22B	108.2
C12—C4—C3	117.09 (16)	H22A—C22—H22B	107.3
C12—C4—C5	119.12 (17)	C22—C23—C24	108.8 (2)
C3—C4—C5	123.79 (17)	C22—C23—C25	111.58 (19)
C6—C5—C4	121.29 (18)	C24—C23—C25	113.3 (2)
C6—C5—H5	119.4	C22—C23—H23	107.7
C4—C5—H5	119.4	C24—C23—H23	107.7
C5—C6—C7	121.13 (17)	C25—C23—H23	107.7
C5—C6—H6	119.4	C23—C24—H24A	109.5
C7—C6—H6	119.4	C23—C24—H24B	109.5
C8—C7—C11	117.09 (17)	H24A—C24—H24B	109.5
C8—C7—C6	123.87 (17)	C23—C24—H24C	109.5
C11—C7—C6	119.03 (17)	H24A—C24—H24C	109.5
C9—C8—C7	119.28 (18)	H24B—C24—H24C	109.5
C9—C8—H8	120.4	C23—C25—H25A	109.5
C7—C8—H8	120.4	C23—C25—H25B	109.5
C8—C9—C10	119.52 (19)	H25A—C25—H25B	109.5
C8—C9—H9	120.2	C23—C25—H25C	109.5
C10—C9—H9	120.2	H25A—C25—H25C	109.5
N2—C10—C9	123.20 (18)	H25B—C25—H25C	109.5
N2—C10—H10	118.4	C1—N1—C12	118.00 (15)
C9—C10—H10	118.4	C1—N1—Ni1	128.79 (12)
N2—C11—C7	123.15 (15)	C12—N1—Ni1	113.20 (10)
N2—C11—C12	117.06 (14)	C10—N2—C11	117.69 (15)
C7—C11—C12	119.79 (15)	C10—N2—Ni1	129.60 (12)
N1—C12—C4	122.96 (15)	C11—N2—Ni1	112.70 (10)
N1—C12—C11	117.43 (14)	C20—N3—C19	112.25 (14)
C4—C12—C11	119.61 (15)	C20—N3—Ni1	108.84 (10)
O1—C13—C14	121.15 (16)	C19—N3—Ni1	110.15 (11)
O1—C13—C18	120.38 (16)	C20—N3—H1N3	105.1 (12)
C14—C13—C18	118.48 (16)	C19—N3—H1N3	110.7 (12)
C15—C14—C13	120.5 (2)	Ni1—N3—H1N3	109.6 (12)
C15—C14—H14	119.7	C13—O1—Ni1	117.47 (9)
C13—C14—H14	119.7	C21—O2—Ni1	116.85 (10)
C16—C15—C14	120.8 (2)	Ni1—O4—H1O4	114.3
C16—C15—H15	119.6	Ni1—O4—H2O4	107.9
C14—C15—H15	119.6	H1O4—O4—H2O4	112.2
C15—C16—C17	118.98 (19)	H1O5—O5—H2O5	98.5
C15—C16—H16	120.5	H1O6—O6—H2O6	108.0
C17—C16—H16	120.5	H1O7—O7—H2O7	127.2
C16—C17—C18	121.7 (2)	H1O8—O8—H2O8	111.9
C16—C17—H17	119.1	H1O9—O9—H2O9	102.6
C18—C17—H17	119.1	O2—Ni1—O1	91.63 (5)
C17—C18—C13	119.40 (17)	O2—Ni1—N1	171.46 (5)
C17—C18—C19	121.50 (17)	O1—Ni1—N1	95.60 (5)
C13—C18—C19	119.03 (16)	O2—Ni1—N2	93.90 (5)
N3—C19—C18	110.88 (14)	O1—Ni1—N2	171.73 (5)
N3—C19—H19A	109.5	N1—Ni1—N2	79.43 (5)
C18—C19—H19A	109.5	O2—Ni1—O4	95.06 (5)
N3—C19—H19B	109.5	O1—Ni1—O4	89.82 (5)
C18—C19—H19B	109.5	N1—Ni1—O4	89.52 (5)
H19A—C19—H19B	108.1	N2—Ni1—O4	83.59 (5)
N3—C20—C21	109.33 (13)	O2—Ni1—N3	80.50 (5)
N3—C20—C22	109.57 (14)	O1—Ni1—N3	90.78 (6)
C21—C20—C22	111.54 (15)	N1—Ni1—N3	94.83 (5)
N3—C20—H20	108.8	N2—Ni1—N3	96.19 (6)
C21—C20—H20	108.8	O4—Ni1—N3	175.53 (5)
			
N1—C1—C2—C3	−0.2 (3)	C7—C11—N2—C10	3.3 (2)
C1—C2—C3—C4	−1.3 (3)	C12—C11—N2—C10	−176.12 (16)
C2—C3—C4—C12	1.1 (3)	C7—C11—N2—Ni1	−177.88 (13)
C2—C3—C4—C5	−178.9 (2)	C12—C11—N2—Ni1	2.74 (17)
C12—C4—C5—C6	−1.3 (3)	C21—C20—N3—C19	−97.20 (16)
C3—C4—C5—C6	178.7 (2)	C22—C20—N3—C19	140.27 (15)
C4—C5—C6—C7	0.2 (4)	C21—C20—N3—Ni1	25.00 (16)
C5—C6—C7—C8	−177.7 (2)	C22—C20—N3—Ni1	−97.53 (15)
C5—C6—C7—C11	1.3 (3)	C18—C19—N3—C20	56.96 (19)
C11—C7—C8—C9	−0.5 (3)	C18—C19—N3—Ni1	−64.49 (16)
C6—C7—C8—C9	178.5 (2)	C14—C13—O1—Ni1	125.71 (15)
C7—C8—C9—C10	1.7 (3)	C18—C13—O1—Ni1	−54.29 (19)
C8—C9—C10—N2	−0.4 (4)	O3—C21—O2—Ni1	−162.77 (14)
C8—C7—C11—N2	−2.0 (3)	C20—C21—O2—Ni1	20.04 (18)
C6—C7—C11—N2	178.93 (17)	C21—O2—Ni1—O1	86.89 (11)
C8—C7—C11—C12	177.34 (16)	C21—O2—Ni1—N1	−60.9 (4)
C6—C7—C11—C12	−1.7 (3)	C21—O2—Ni1—N2	−99.25 (11)
C3—C4—C12—N1	0.4 (3)	C21—O2—Ni1—O4	176.86 (11)
C5—C4—C12—N1	−179.63 (17)	C21—O2—Ni1—N3	−3.62 (11)
C3—C4—C12—C11	−179.14 (16)	C13—O1—Ni1—O2	−43.27 (13)
C5—C4—C12—C11	0.9 (3)	C13—O1—Ni1—N1	132.18 (12)
N2—C11—C12—N1	0.5 (2)	C13—O1—Ni1—N2	−175.3 (3)
C7—C11—C12—N1	−178.91 (15)	C13—O1—Ni1—O4	−138.32 (12)
N2—C11—C12—C4	−179.97 (15)	C13—O1—Ni1—N3	37.25 (12)
C7—C11—C12—C4	0.6 (2)	C1—N1—Ni1—O2	143.3 (3)
O1—C13—C14—C15	179.01 (18)	C12—N1—Ni1—O2	−35.3 (4)
C18—C13—C14—C15	−1.0 (3)	C1—N1—Ni1—O1	−4.37 (17)
C13—C14—C15—C16	−0.2 (3)	C12—N1—Ni1—O1	177.07 (12)
C14—C15—C16—C17	1.4 (3)	C1—N1—Ni1—N2	−177.71 (17)
C15—C16—C17—C18	−1.4 (3)	C12—N1—Ni1—N2	3.73 (12)
C16—C17—C18—C13	0.2 (3)	C1—N1—Ni1—O4	−94.14 (17)
C16—C17—C18—C19	177.10 (19)	C12—N1—Ni1—O4	87.30 (12)
O1—C13—C18—C17	−179.03 (16)	C1—N1—Ni1—N3	86.88 (17)
C14—C13—C18—C17	1.0 (3)	C12—N1—Ni1—N3	−91.68 (12)
O1—C13—C18—C19	4.0 (2)	C10—N2—Ni1—O2	−10.16 (17)
C14—C13—C18—C19	−175.97 (17)	C11—N2—Ni1—O2	171.16 (11)
C17—C18—C19—N3	−114.01 (19)	C10—N2—Ni1—O1	121.7 (4)
C13—C18—C19—N3	62.9 (2)	C11—N2—Ni1—O1	−57.0 (4)
N3—C20—C21—O3	151.94 (15)	C10—N2—Ni1—N1	175.22 (17)
C22—C20—C21—O3	−86.71 (19)	C11—N2—Ni1—N1	−3.47 (11)
N3—C20—C21—O2	−30.7 (2)	C10—N2—Ni1—O4	84.50 (17)
C22—C20—C21—O2	90.65 (18)	C11—N2—Ni1—O4	−94.18 (11)
N3—C20—C22—C23	169.60 (16)	C10—N2—Ni1—N3	−91.01 (17)
C21—C20—C22—C23	48.4 (2)	C11—N2—Ni1—N3	90.31 (11)
C20—C22—C23—C24	−172.7 (2)	C20—N3—Ni1—O2	−13.11 (11)
C20—C22—C23—C25	61.5 (2)	C19—N3—Ni1—O2	110.36 (11)
C2—C1—N1—C12	1.6 (3)	C20—N3—Ni1—O1	−104.63 (12)
C2—C1—N1—Ni1	−176.86 (15)	C19—N3—Ni1—O1	18.84 (11)
C4—C12—N1—C1	−1.7 (3)	C20—N3—Ni1—N1	159.69 (11)
C11—C12—N1—C1	177.78 (16)	C19—N3—Ni1—N1	−76.85 (11)
C4—C12—N1—Ni1	176.99 (13)	C20—N3—Ni1—N2	79.83 (12)
C11—C12—N1—Ni1	−3.49 (18)	C19—N3—Ni1—N2	−156.70 (11)
C9—C10—N2—C11	−2.0 (3)	C20—N3—Ni1—O4	−7.0 (9)
C9—C10—N2—Ni1	179.36 (16)	C19—N3—Ni1—O4	116.5 (8)

### Hydrogen-bond geometry (Å, º)

**Table:**

*D*—H···*A*	*D*—H	H···*A*	*D*···*A*	*D*—H···*A*
O4—H1*O*4···O7	0.83	1.89	2.709 (2)	169
O4—H2*O*4···O5	0.98	1.80	2.772 (2)	169
O5—H1*O*5···O3^i^	1.00	1.82	2.8137 (19)	171
O5—H2*O*5···O1	0.95	1.81	2.7393 (19)	164
O6—H1*O*6···O2	0.96	1.83	2.7310 (18)	156
O6—H2*O*6···O9	1.00	1.85	2.807 (2)	160
O7—H1*O*7···O8	0.93	1.78	2.693 (3)	171
O7—H2*O*7···O6	0.94	1.91	2.832 (3)	165
O8—H1*O*8···O6^ii^	0.87	1.89	2.730 (3)	162
O8—H2*O*8···O5^ii^	1.08	1.68	2.749 (2)	169
O9—H1*O*9···O3	0.95	1.81	2.749 (2)	171
O9—H2*O*9···O1^iii^	0.87	1.98	2.8459 (18)	173

Symmetry codes: (i) −*x*+1, *y*−1/2, −*z*+3/2; (ii) *x*+1/2, −*y*+1/2, −*z*+1; (iii) −*x*+1, *y*+1/2, −*z*+3/2.
